# Reshaping Bioacoustics Event Detection: Leveraging Few-Shot Learning (FSL) with Transductive Inference and Data Augmentation

**DOI:** 10.3390/bioengineering11070685

**Published:** 2024-07-05

**Authors:** Nouman Ijaz, Farhad Banoori, Insoo Koo

**Affiliations:** 1 Department of Electrical, Electronics and Computer Engineering, University of Ulsan, Ulsan 44610, Republic of Korea; noumanijaz341@gmail.com; 2 School of Electronics and Information Engineering, South China University of Technology, Guangzhou 510641, China; farhadscut@gmail.com; 3 Faculty of Computer Sciences, Department of Computer Science, ILMA University, Karachi City 74900, Pakistan

**Keywords:** few-shot learning (FSL), bioacoustics event detection, transductive inference, data augmentation

## Abstract

Bioacoustic event detection is a demanding endeavor involving recognizing and classifying the sounds animals make in their natural habitats. Traditional supervised learning requires a large amount of labeled data, which are hard to come by in bioacoustics. This paper presents a few-shot learning (FSL) method incorporating transductive inference and data augmentation to address the issues of too few labeled events and small volumes of recordings. Here, transductive inference iteratively alters class prototypes and feature extractors to seize essential patterns, whereas data augmentation applies SpecAugment on Mel spectrogram features to augment training data. The proposed approach is evaluated by using the Detecting and Classifying Acoustic Scenes and Events (DCASE) 2022 and 2021 datasets. Extensive experimental results demonstrate that all components of the proposed method achieve significant F-score improvements of 27% and 10%, for the DCASE-2022 and DCASE-2021 datasets, respectively, compared to recent advanced approaches. Moreover, our method is helpful in FSL tasks because it effectively adapts to sounds from various animal species, recordings, and durations.

## 1. Introduction

Detection and interpretation of bioacoustic events are vital in wildlife monitoring, marine life health monitoring, multimedia indexing, and audio surveillance [1]. These tasks are challenging due to the scarcity of annotated data, as well as class imbalances, noisy environments, and the need for specialized skills to annotate the data. Deep learning has succeeded in recent years by utilizing a vast amount of labeled data for training. Many approaches have been introduced, resulting in good accuracy. However, accuracy can suffer from insufficient amounts of labeled data. Humans can recognize a new class by looking at only a few samples, or even just one. For instance, children can generalize about pandas based on a single image, or will hear about pandas from other children. From that perspective, few-shot learning (FSL) is a promising strategy that learns from small amounts of labeled data. It has emerged as a solution to the aforementioned problems [2,3], and is analogous to the way the human brain functions [4].

In the current age of information technology, there is a lot of unstructured data available, but it can be hard to find annotated data [5]. Bioacoustic event detection determines whether (and when) a specific animal vocalization occurs in an audio recording [6]. To start, it can be challenging to record vocalizations of some species; furthermore, only people with specialized skills can annotate the obtained data, which is a labor-intensive process. In that case, few-shot (FS) bioacoustic event detection has arisen as a novel field of study into annotating lengthy recordings [7]. Additionally, widely recognized methods build on standard supervised learning with regard to effectiveness in other sound event detection (SED) scenarios, which might not deliver the same performance in this particular task. It is reasonable to claim that FS bioacoustic event detection is interesting for research because it can meet the demand.

### 1.1. Related Work

FS bioacoustic event detection is a task that involves identifying the onset and termination of acoustic events in a series of audio recordings obtained from natural environments. In recent studies, the acoustic characteristics of advanced core structures have been shown to significantly impact the vibroacoustic performance of sandwich systems enhancing the overall sound transmission loss (STL) of the system [8]. Researchers have investigated how bioacoustics presents unique challenges that make it a particularly demanding application domain for SED [9,10]. The bioacoustic environment is surprising and varied. Species, noises, and recording circumstances can affect acoustic signatures considerably. Earlier methods focused on manually extracting features and using basic signal processing methods, which worked well in controlled situations [11]. However, those methods had trouble with the complexity and variability of bioacoustic environments in the real world. With the emergence of deep learning, there has been a significant change in the way we approach data analysis. Convolutional neural networks (CNNs) and recurrent neural networks (RNNs) have proven highly effective in detecting complex patterns in bioacoustic data [12]. Nevertheless, these methods often need large quantities of labeled data, thus posing a significant challenge to the time-consuming process of obtaining annotated bioacoustic recordings and to the poor generalizations that result from a lack of supervised data [10,11,12,13,14,15,16,17].

Researchers have proposed FSL as a solution to the problem of training with small amounts of labeled data [18]. An FSL classifier can potentially detect novel classes not present in the training set with just a few samples from each new class. It has been observed that SED only locates the onset and termination of certain sounds of interest, but FSL can identify a new class with just a few labeled samples by fusing the concept with SED [19,20]. N-way K-shot classification is typically used in FSL studies [20], where N is the number of classes, and K is the number of known samples for each class. The problem of bioacoustic event detection has received a lot of attention, and datasets have been released every year from the Detection and Classification of Acoustic Scenes and Events (DCASE) community. Different methods have been used to handle FSL problems. Some compute embeddings (learned representation spaces) to help distinguish between classes not seen before by using prior information about similarities between sounds [21,22]. A baseline system uses a prototypical network for FS SED detection, which works on computing Euclidean distance between class prototypes [18]. But due to background noise and acoustic interference, the class prototypes obtained from such a support set may not precisely represent the class center.

Recent studies demonstrated that it is still insufficient to use a simple prototypical network for a small-scale support set to correctly imply the class center [23]. Environmental audio recordings contain a lot of background noise; per-channel energy normalization (PCEN) techniques are mostly applied to improve the quality of the data [24]. This technique involves normalizing the energy of each channel, Gaussianizing the magnitude, and de-correlating the frequency band. However, the most essential factor in the performance of FS bioacoustic event detection is feature extraction. In this process, the most suitable audio features for detection of acoustic events are the time–frequency representation known as a Mel spectrogram and extracted from raw audio data [25,26]. Furthermore, in most of the previous research, only Mel spectrograms were utilized or a fine-tuned model overfits the support set. At the same time, other vital features were not investigated [27]. Additionally, the potential of semi-supervised techniques and various loss functions remains untapped. Overall, all the issues regarding data scarcity, environmental variability, environmental conservation, and overfitting challenges provide significant motivation to explore and present a non-intrusive, scalable, diverse, and comprehensive bioacoustic event detection approach.

### 1.2. Motivation

The motivation for using transductive inference for sound event detection is that it more effectively represents class prototypes in a few-shot setting compared to other methods, such as inductive learning. More specifically, transductive inference uses a large amount of unlabeled data that is often available in many real-world applications to improve the model’s performance. To create new training examples, data augmentation is a process that transforms the original data. It helps a model learn more robust representations, and improves generalizations about new and unseen data. The combination of data augmentation and transductive inference was motivated by the fact that they complement each other and can lead to even better performance in SED tasks with a limited amount of labeled data. Data augmentation increases diversity in the labeled data, while transductive inference helps the model better utilize the unlabeled data to improve representations of class prototypes. A Mel spectrogram with PCEN is adopted as an input feature due to its excellent performance in most audio processing tasks.

### 1.3. Contribution

This article details the problems of FS bioacoustic event detection and proposes an effective method for solving them. The major contributions of this research are outlined below:

1. We investigate, examine, and comprehend audio feature extraction. We use an efficient and unique FSL technique to detect the onset and termination of a bioacoustic event in the DCASE-2022 and DCASE-2021 audio datasets. Moreover, a Mel spectrogram with PCEN is applied to the audio features, which yields higher performance in acoustic event detection.
2. We calculate cross-entropy loss between predicted and actual labels, and then use back-propagation to update model parameters. The frozen backbone model is utilized as the feature extractor. Then, a classifier with parameterized prototypes for each class in the support set is built, which leads to greater accuracy than from the baseline system. Hence, we provide an innovative approach to fine-tune the feature extractor and make it task-specific.

The subsequent sections of this paper are structured in the following manner: Section 2 illustrates the proposed methodology regarding few-shot bioacoustic event detection. It includes all significant mathematical modeling and computations related to the proposed technique. Section 3 describes the experimental setup, the datasets, and the performance metrics to illustrate the proposed methodology more effectively. It presents the results used to validate the simulation and analysis, as well as to compare the suggested method to other advanced approaches. Finally, conclusions are drawn in Section 4.

## 2. Our Methodology

**Figure 1:**
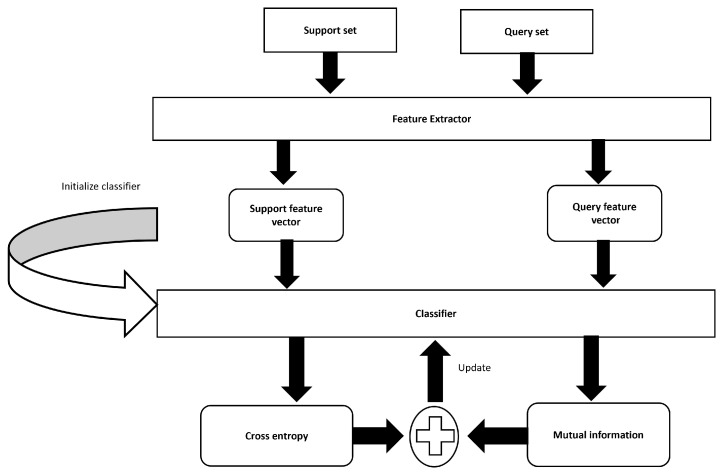
Proposed method of transductive inference.

This section elaborates on the FS bioacoustic event detection method employing transductive inference with data augmentation. Figure 1 illustrates our proposed method that employs transductive inference by leveraging both labeled support sets and unlabeled query sets. The Mel spectrogram is used as an input feature that gives our model access to sound data. PCEN is then applied to the Mel spectrogram, which reduces the dynamic range and normalizes the energy level across channels. Moreover, PCEN reduces background noise and improves robustness to channel distortion. The transformed features are then fed into a transductive learning framework, which increases the mutual information between the features of the labeled training data and unlabeled test data. The stages involved in the suggested method are subdivided into their individual components below.

(**a**)  **Features Extraction**

**Figure 2:**
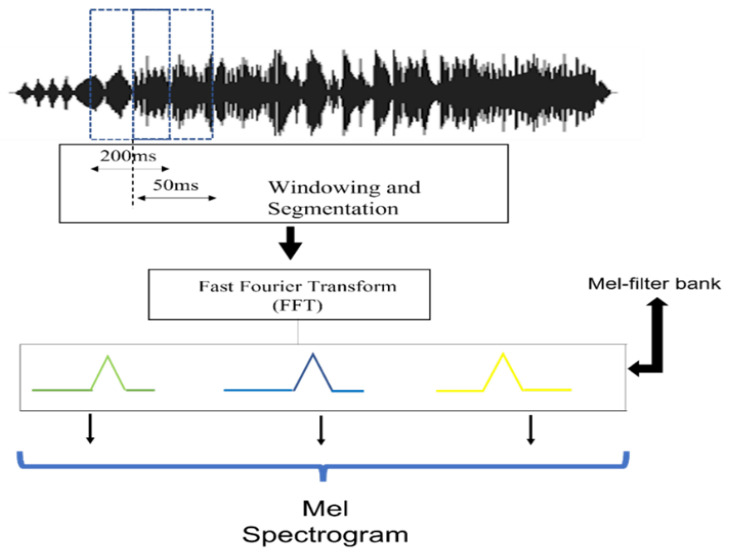
Mel spectrogram extraction from raw audio.

This segment takes a different path compared to traditional methodologies. We concentrate on extracting raw audio features, incorporating contextual and temporal characteristics. This enriches the FSL feature set because a careful approach is essential in bioacoustics, where the intricacies of sound can reflect important ecological events or behaviors. Here, the Mel spectrogram with PCEN is used and features are extracted using Python’s librosa package. The function librosa.load puts the audio signal into memory and returns it as a two-dimensional NumPy array along with the sample rate. After that, computing a short-time Fourier transform (STFT) of the audio signal results in the NumPy array representing the audio signal spectrogram. The resulting complex valued spectrogram is then converted to the Mel frequency domain by applying a Mel filterbank, which maps the linear frequency bins to Mel frequency bins. Table 1 lists the parameters in this research to extract a Mel spectrogram. After the Mel spectrogram is created, PCEN is applied to reduce background noise and improve robustness to channel distortion, which upgrades the performance of the acoustic event detection task. Figure 2 shows the Mel spectrogram feature extraction process in this research.

The original datasets were resampled to a fixed sample rate of 22.05 kHz, and the major parameters are listed in Table 1. Segment length is the length of each audio segment processed to extract the Mel spectrogram. Hop size, known as stride or step size, determines the overlap between consecutive frames or segments of a signal. The size of the fast Fourier transform (FFT) applied to the audio segments is denoted by n_fft and n_Mel is the number of Mel frequency bins used to represent the audio signal. The Mel scale is a non-linear frequency scale designed to better reflect the human perception of sound. The size of the hops in the Mel frequency bins is denoted with hop_Mel and eps denotes the value used as a small constant to prevent division by zero when calculating PCEN, which helps maintain a consistent and non-zero energy level across different audio segments, even in the presence of low-level noise. Thus, eps serves as the lower bound of the Mel spectrum to prevent log 0.

After feature extraction, it is necessary to carefully examine the shape of the input features fed into the backbone network for training. There were 101,702 and 17,168 training samples taken from the DCASE-2022 and DCASE-2021 datasets, respectively, and 392,182 and 284,136 samples, respectively, for evaluation.

(**b**)  **Data Augmentation**

**Figure 3:**
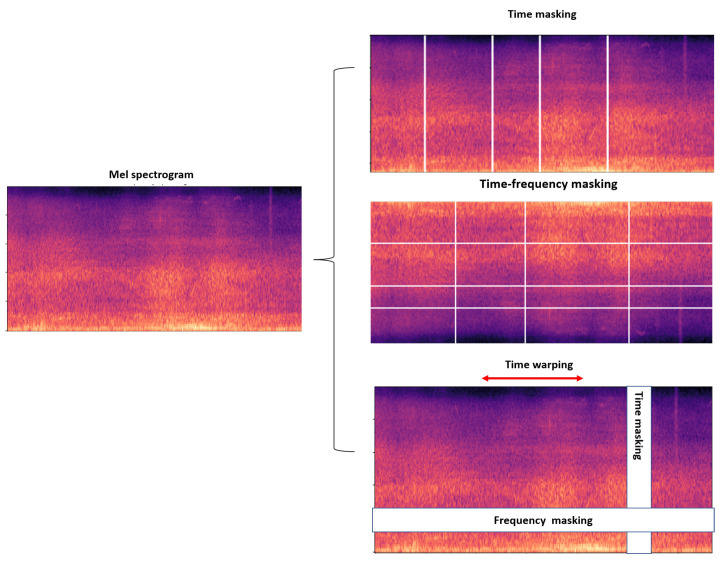
Data augmentation (specAugment).

There are two types of data augmentation techniques, i.e., raw data augmentation and SpecAugment [28,29]. Raw data augmentation applies transformations such as pitch shift and noise injection to the audio waveform. Meanwhile, SpecAugment operates on the spectrogram representation of the audio data to enhance the target model’s robustness and provide better class prototype representation. SpecAugment is effective in acoustic event detection tasks by introducing variations and enhancing the diversity of the training data. The operations in this method include time masking, frequency masking, and time warping, shown in Figure 3.

**Time Masking:** Time masking entails choosing a random time segment $\left[t_{0},t_{0}+t\right]$ in the spectrogram and setting the values to zero or another constant. The parameter *t* is generated randomly from a discrete uniform distribution between 0 and a maximum time mask parameter $t_{\max}$. This makes the model insensitive to minor changes in time and thus enhances the model’s ability to handle abrupt changes.

**Frequency Masking:** Frequency masking is similar to time masking but is applied along the frequency axis and is used to reduce high-frequency noise. The random frequency band of $\left[f_{0},f_{0}+f\right]$ is selected and its values are concealed. The function *f* is derived randomly from a uniform distribution over the range of 0 and a maximum frequency mask parameter $f_{\max}$. This leads to a model that is less sensitive to small shifts in frequency, thus improving the model’s ability to generalize.

**Time Warping:** Time warping involves stretching or compressing the time axis of the spectrogram. A random anchor point is selected along the time axis, and a random warp factor is applied to the left and right of this point. The warping process displaces the spectrogram values to emulate minor speed changes in the audio to assist the model in recognizing temporal distortions. These techniques in aggregate increase the raw number of inputs seen by the model and make the training data more varied, thereby increasing the model’s stability and applicability.

(**c**)  **Backbone Building**

**Figure 4:**
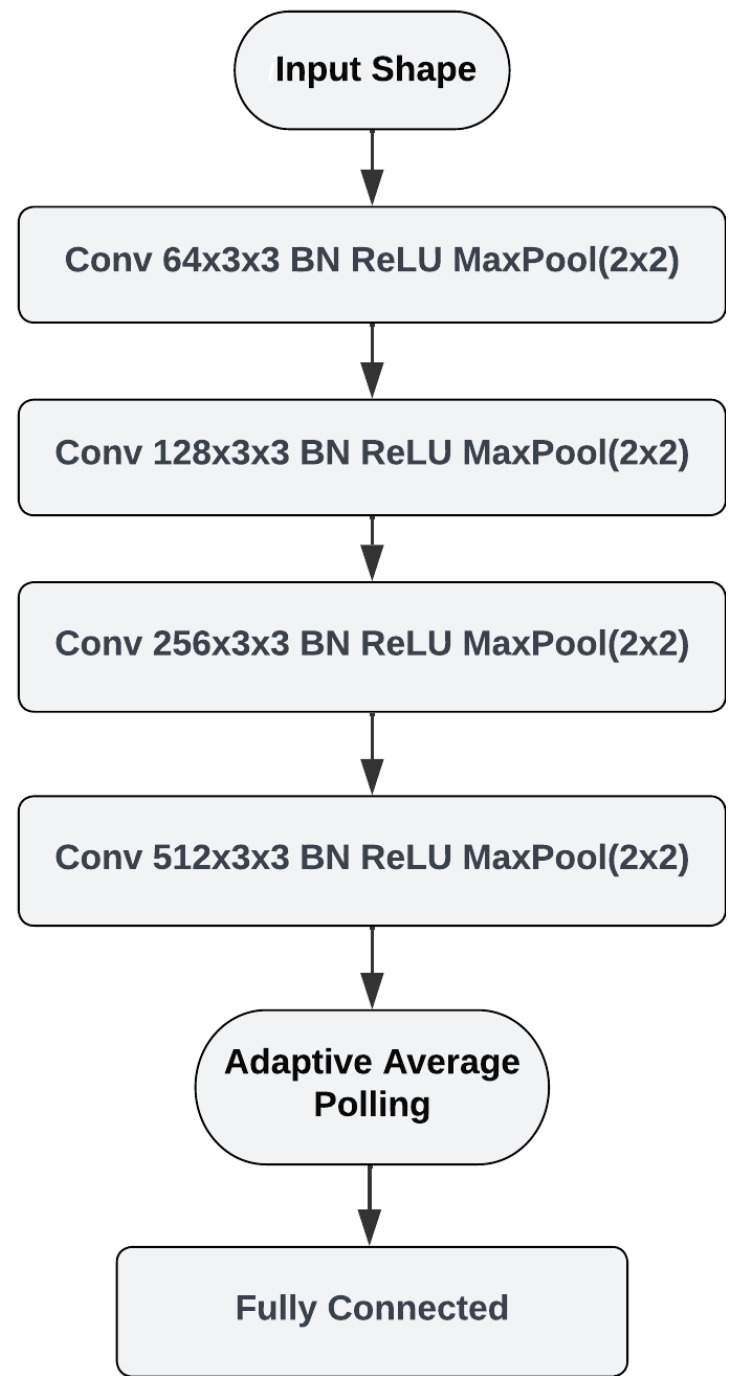
Architecture of the backbone model.

In FSL, the goal is to learn a model that can quickly adapt to new tasks with only a few labeled examples. A common approach in FSL is to use a backbone model pre-trained on a large dataset and then fine-tune it on the FSL task [30]. Choosing a backbone model for the FSL task significantly impacts the model’s performance [31]. To classify bioacoustic events in the FS setting, we designed a prototypical network using a four-layer CNN architecture. Figure 4 shows the architecture of the backbone model. The input for our network is the Mel spectrogram extracted from an audio recording. The CNN architecture consists of four convolutional layers with number of filters ranging from 64 to 512. The initial learning rate was adjusted to 0.001, the scheduler gamma to 0.5, and the step size was set to update the learning rate every five epochs; a batch size = 64 was chosen because it is the right choice for the server’s GPU. The Adam optimizer with a loss function for cross-entropy and mutual information is used in the model, which is trained by varying the number of epochs between 5 and 30 for the best performance. Batch normalization is applied after each convolutional layer to normalize the output and improve training performance. MaxPooling is applied after each convolutional layer to downsample the feature maps. Average pooling is then applied to obtain the class prototypes.

**Transductive Learning:** The purpose of transductive learning is to create predictions for certain test cases that are already known, as opposed to making a generalization that applies to all possible test examples [32]. Here, the model is trained on a certain subset of data points that have been labeled, and then, it is given the task of predicting labels in a subset of data points that have not been labeled. We have a support set (S) and a query set (Q) for a specific FS task: $S=\{X_{s},Y_{s}\}$ and $Q=\{X_{q},Y_{q}\}$. We denote the feature extractor as $f_{\theta }:X\to Z\in R^{d}$, and *Z* expresses the set of extracted features. A base dataset, $D_{\mathrm{base}}=\left\{X_{\mathrm{base}},Y_{\mathrm{base}}\right\}$, is given to pre-train the feature extractor. This dataset contains numerous labeled examples from various classes and provides a robust initialization for the model’s parameters, ensuring the feature extractor learns generalizable features before being fine-tuned on the few-shot learning task. The construction principle of the support set (S) is to select a small subset of labeled data points from the target class representing categories that need to be learned. It provides the labeled examples needed for the model to adapt to new, previously unseen classes. The query set (Q) contains unlabeled examples from the same target classes as the support set (S). Transductive learning aims to improve the model’s accuracy by leveraging both the labeled support set (S) and the unlabeled query set (Q). During the transductive inference process, the model iteratively refines its predictions by considering the entire distribution of the query set. This approach enhances the model’s ability to generalize from the few-shot support set to the unlabeled query set, thereby improving performance in cases with limited labeled data.

During the inference process, a technique known as transductive information maximization (TIM) increases the level of mutual information between the query instances and their projected labels [33]. This approach is exceptional because it optimizes the mutual information between labeled and unlabeled data within a transductive framework. At this vital stage, the focus is on enhancing the correlation between data projections and labels, thereby utilizing the underlying data structure to its fullest potential. This involves not only the labeled dataset but incorporating unlabeled data. Here, transductive inference, which takes into consideration the entire data distribution, allows TIM to increase model generalizations about previously unknown data, going beyond traditional learning models. Moreover, iterative refinement entails continuously updating pseudo-labels for the unlabeled data and enhancing mutual information with each iteration until the model achieves optimal performance.

Specifically, various backbone networks underwent training using the standard supervised learning approach with the training data provided by the DCASE task organizers, and then we employed standard supervised learning. Here, cross-entropy loss is calculated between the predicted label and actual labels, and back-propagation is used to update the model parameters. A classifier is parameterized, $W=\left[w_{1},\ldots ,w_{k}\right]\in R^{K\times d}$, and its parameters are initialized with the prototypes of each class in the support set. The backbone model is frozen and employed as a feature extractor. Regarding labels given to the features, posterior distribution is prescribed as follows:

(1) $$p_{ik}=P\left(Y=k|X=x_{i};W,\theta \right)$$

Correspondingly, the marginal distribution across query labels is outlined:

(2) $${\hat{p}}_{k}=P\left(Y=k;W,\theta \right)$$

$p_{ik}$ and ${\hat{p}}_{k}$ are calculated:

(3) $$p_{ik}=\frac{exp^{w_{k}\cdot z_{i}}}{\sum_{c=1}^{K}expw_{c}\cdot z_{i}},{\hat{p}}_{k}=\frac{1}{\left|Q\right|}\sum_{i=Q}p_{ik}$$

Subsequently, the classifier is fine-tuned by employing the loss function:

(4) $$L_{w}=\lambda_{CE}\cdot CE-I\left(Y_{Q};X_{Q}\right)$$

Equation (4) considers both mutual information loss and conventional cross-entropy loss on the support set:

(5) $$I\left(Y_{Q};X_{Q}\right)=-\sum_{k=1}^{K}\left({\hat{p}}_{k}\right)\log\left({\hat{p}}_{k}\right)+\frac{1}{\left|Q\right|}\sum_{i\in Q}\sum_{k=1}^{K}p_{ik}\log\left(p_{ik}\right)$$

(6) $$CE=-\frac{1}{\left|S\right|}\sum_{i\in S}\sum_{i=1}^{K}y_{ik}\log\left(p_{ik}\right)$$

in which $y_{ik}$ and $p_{ik}$ represent the true label of the sample within the support set and the prediction result, respectively. Thus, $\lambda_{CE}$ is a hyper-parameter, and we set $\lambda_{CE}=1$.

## 3. Experiment

In this section, we introduce the datasets used in this work, and present the experimental setup and results.

### 3.1. Experimental Datasets

- **DCASE-2021**

The DCASE-2021 development datasets consist of predetermined training and validation sets for system development and contain audio recordings from multiple sources with associated annotations in a task-specific format. Each recording in the training set has multiple-class temporal annotations that are positive (POS), negative (NEG), and unknown (UNK). In contrast, each recording in the validation set only has a single-class temporal annotation (POS or UNK). Table 2 lists the recording devices utilized, the number of audio files, the total length of the set, the number of labels, and the number of annotated events.

- **DCASE-2022**

The DCASE-2022 datasets, like DCASE-2021, include predetermined sets of training and validation samples for system development. DCASE-2022 presents a superior source than its 2021 counterpart due to its substantially increased size, featuring more than twice as many audio samples. Furthermore, it incorporates more than double the variety of acoustic scenes and sound event classes. Both datasets contain recordings in WAV format. Annotation files with the CSV extension have the same name as their corresponding audio files. Multi-class annotations are provided for the training set, with positive (POS), negative (NEG), and unknown (UNK) values for samples in each class. Conversely, single-class (interest) annotations are presented, with positive (POS) and unknown (UNK) for samples in the validation set. These recordings were gathered from various bioacoustic sources, such as birds, spotted hyenas, jackdaws, meerkats, and wetlands birds from all over the world. Table 3 gives a summary of the DCASE-2022 datasets.

In FS bioacoustic event detection datasets, the validation set comprises recordings of both mammals and birds. These recordings came from various places, such as research projects, public databases, and citizen science projects. Summaries are given below.

- **Hyenas (HT, HV)**:

Spotted hyenas are social animals that communicate with each other. They live in groups that can break up and come back together over time. Mark Johnson and Frants Jensen used custom-made audio tags built into GPS/acoustic collars to record the sounds these animals make [34]. Kay Holekamp, a famous ecologist, put collars on female hyenas from a group of spotted hyenas (the Talek west clan) in Kenya’s Masai Mara as part of a study on how different species communicate and act as a group [35]. The hyena vocalizations among the recordings used for this task were categorized into different types based on what was known about the hyena vocal repertoire. Hyena recordings and their annotations from the HT subset were used as the development set, and those from the HV subset were used as the validation set. The vocalizations in both sets are distinct from one another.

- **Polish Baltic sea birds (PB):**

The PB dataset consists of six 30-minute recordings of bird flight calls along the Polish Baltic Sea coast [36].The recordings were obtained during the fall migration seasons of 2016, 2017, and 2018 by using three autonomous recording units equipped with Song Meters SM2 from Wildlife Acoustics, Inc. That company focuses on developing and selling bioacoustic monitoring systems and software. The recording units were placed in different locations (near a lake, on a dune, and in a forest clearing) to provide diverse acoustic backgrounds. The PB dataset includes flight calls by two passerine bird species, the song thrush (Turdus philomelos) and the blackbird (Turdus merula), with three recordings per species. The flight calls range in duration from 8 to 400 milliseconds and have a fundamental frequency range of 5–9 kHz, which is typical for the selected species. The PB dataset was annotated by Hanna Pamula, a researcher who manually identified and labeled the passerine night flight calls in the recordings [37]. The dataset is part of the development set used for validation. The PB dataset is a valuable resource for researchers interested in studying bird migration patterns and behaviors and who are developing automated methods for detecting and classifying bird flight calls.

- **HumBug (HB):**

The HB dataset contains acoustic recordings of mosquitoes captured in the wild in Thailand, plus Culex quinquefasciatus (the southern house mosquito, a major disease transmitter) lab-cultured in Oxford, U.K. [38]. Mosquitoes produce sounds as a byproduct of flight and as a means of communication and for mating, with fundamental frequencies ranging from 150 to 750 Hz. The recordings in this challenge are sub-datasets named OxZoology and Thailand [38]. The acoustic data were recorded with a high-specification field microphone (the Telinga EM-23) coupled with an Olympus LS-14 recorder. The insects were placed in plastic containers, and sounds were recorded using the field microphone in close proximity. The HumBug dataset [38] is part of the development set used for validation and can be utilized to develop and test machine learning models to automatically detect and classify mosquito sounds. This dataset is valuable for researchers interested in understanding mosquito behavior and ecology and who are developing tools for mosquito control and monitoring of mosquito-borne diseases.

- **The Meerkats (MT, ME):**

This dataset consists of various vocalizations produced by meerkats, a social mongoose species [39]. The dataset focuses on pup-begging vocalizations, which are short, high-pitched peeps. Recordings were obtained during the breeding season at the Kalahari Meerkat Project using AudioMoth recorders placed in meerkat burrows, and annotations were made by Joe Morford [40]. The dataset is part of the development set for validation and can be used to develop and test machine learning models for automatic detection and classification of meerkat vocalizations. It is useful for studying meerkat communication and behavior and in developing tools for studying social animal populations [41].

### 3.2. Data Preprocessing

Getting the data in order before starting an experiment is necessary. The first thing that needed to be accomplished was to transmit all the DCASE-2022 and DCASE-2021 development datasets to the server. After sending the datasets, it was noted that not all audio recordings had the same sampling rate. Therefore, we decide to downsample the raw audio to 22.05 kHz for simplicity and efficient training of the network.

#### 3.2.1. Experimental Setup

- **Software**

All programming in this project was performed with the Visual Studio Code (VS Code) IDE and Python version 3.10. Pandas, NumPy, and Matplotlib were among the libraries utilized in the tests for data processing. The Sound File library was used to read and write audio files, while Python’s librosa handled Mel spectrogram feature extraction. An open-source framework, Hydra, was used to develop and run experiments in PyTorch. The main function of the Hydra framework is to enable execution of a hierarchical setup for customization through configuration files and command line overrides. Moreover, libraries such as tqdm were used to display a progression bar whenever the data were processed. While interfacing with the server, Xshell was utilized, and the Xftp 7 platform transmitted all data to the server.

- **Hardware**

The following is a list of the hardware used. The computer in the laboratory had an I-5 CPU, 8 GB of RAM, with Windows 10 Pro as its operating system. A server that is used on the training data needs significant processing power. Ours employed a sixth-generation Intel processor with three 1080 TI Nvidia GPUs, together with 4 TB of internal storage and a 250 GB solid-state drive.

- **Performance Metrics**

After completing feature generation, model training, and obtaining the output, the subsequent phase involved assessing the model’s effectiveness and efficiency with a test dataset [42]. In the realm of machine learning, each algorithm is assessed based on distinct performance metrics due to the variance in datasets and the characteristics of the events detected. Therefore, traditional metrics based on onset detection do not suffice. Instead, intersection over union (IoU) with a minimum 30% overlap was used to identify potential prediction matches. IoU and bipartite graph matching exclusively to ground truth positive (POS) events and predicted events determined a true positive (TP).

**Figure 5:**
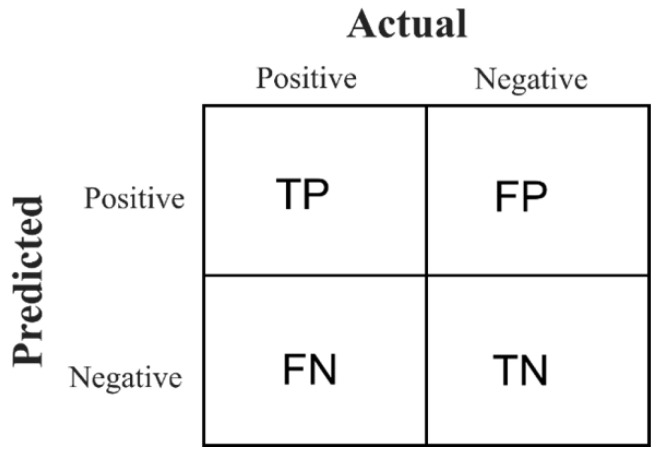
Terminology used in performance metrics.

In an SED task, a TP is defined as a predicted event that matches the ground truth event, and a false positive (FP) is a projected event that did not match any ground truth event. A false negative (FN) measured an unpredicted ground truth event, whereas a true negative (TN) is one in which the model accurately detected and categorized an instance that was not positive (see Figure 5). In this work, ground truth events were POS events from the provided class, and UNK events with some degree of ambiguity. An event-based F-score calculated with the metrics precision and recall was used, which is one of SED’s most widely used performance metrics [43].

(7) $$Precision=\frac{TP}{TP+FP}$$

(8) $$Recall=\frac{TP}{TP+FN}$$

F-score is the harmonic mean of precision and recall giving the same weight:

(9) $$F-score=2\times \frac{Precision\times Recall}{Precision+Recall}$$

#### 3.2.2. Baseline System

Every project requires a baseline system, which serves as the project’s starting point. A baseline system aims to establish a starting point for measuring progress and evaluating changes. In the context of technology and engineering, a baseline system is typically a well-understood, established system that serves as the benchmark for evaluating the performance and effectiveness of a new or innovative system. In this experiment, a baseline system was designed using a prototypical network for FS bioacoustics event detection [44]: a CNN-based model with a four-layer structure. A 3 × 3 convolutional layer takes the input tensor and applies 64 filters to produce 64 feature maps. The feature maps are then passed to batch normalization, and the rectified linear unit (ReLU) activation function applies a non-linear transformation, enabling the network to learn more complex representations. Our system uses a five-shot, five-way FSL approach. The model was trained using the Adam optimizer with an initial learning rate of 0.0001 and a batch size of 64. Additionally, a learning rate scheduler reduced the learning rate by a factor of 0.5 every 10 epochs. After predictions were produced, post-processing removed the events with durations shorter than 60% of the shortest shot provided for that file.

PCEN and the Mel spectrogram were employed to create an input feature, and the results obtained were a 29% F-score for DCASE-2022 and 41% for DCASE-2021. Table 4 shows the baseline results for DCASE-2022 and DCASE-2021.

### 3.3. Simulation Results and Discussion

In this section, we evaluate and assess various approaches on the DCASE-2021 and 2022 datasets for detecting bioacoustic events by utilizing FSL with transductive inference and data augmentation as detailed in Table 5. The metrics for evaluation embody F-score, precision, and recall. For DCASE-2021, the baseline approach obtained an F-score of 41.48%, setting the benchmark for proposed methods to surpass. Cheng et al. [45] applied Mel frequency cepstral coefficients (MFCC) and I-vector aspects for training a prototypical network. The Anderson method [46] utilized raw audio augmentation within a straightforward prototypical network, yielding a lower F-score of 26.24%, well below our baseline, hence manifesting underperformance.

Our method employing transductive inference recorded the highest F-score at 49.85%, marking a notable advancement beyond the baseline. The introduction of the TIM+SpecAugment method further raised the F-score to 51.64%, implying how data augmentation notably boosts performance. Additionally, using training and validation sets recorded with diverse microphone types introduced extra variability. With DCASE-2022, the baseline prototypical network approach logged an F-score of 29.59%, suggesting traditional techniques struggle with efficient acoustic scene and event recognition and classification. The strategy by Li et al. [47] attained an F-score of 47.88%, substantially better than the baseline, illustrating the effectiveness of data augmentation for enhancing a prototypical network’s performance. Xiaoxiao and Long [48] used a continual learning approach to bioacoustic event detection, achieving an F-score of 53.87%.

Our TIM methodology registered an F-score of 54.76%, surpassing the approach by Li et al. Our approach leverages transductive inference to make use of unlabeled data, increasing the model’s ability to generalize, which is a crucial benefit when labeled data are scarce, as is common in the FSL context. Further, by integrating the strengths of transductive inference with data augmentation, we formulated the TIM+SpecAugment technique, which recorded a peak F-score of 56.03%. The output underscores that our proposed strategies exceeded the baseline with both datasets, indicating the potential efficacy in practical applications.

#### 3.3.1. Results per Subset

The effectiveness of our suggested approach is exhibited in Table 6 with subsets of the DCASE datasets: HV and PB from DCASE 2021, and ME, HB, and PB from DCASE 2022, depicting our approach’s results across these distinct subsets.

#### 3.3.2. Results per Audio Recording

Table 7 and Table 8 provide detailed breakdowns of the scores per audio recording from the baseline and our proposed method on DCASE-2022 and DCASE-2021 datasets, respectively. Our proposed method, which utilizes transductive inference with data augmentation, achieved better results with both datasets, compared to the baseline. The improvements suggest that transductive learning techniques that update the feature extractor method are more effective than a simple prototypical network approach to bioacoustic event detection.

**Figure 6:**
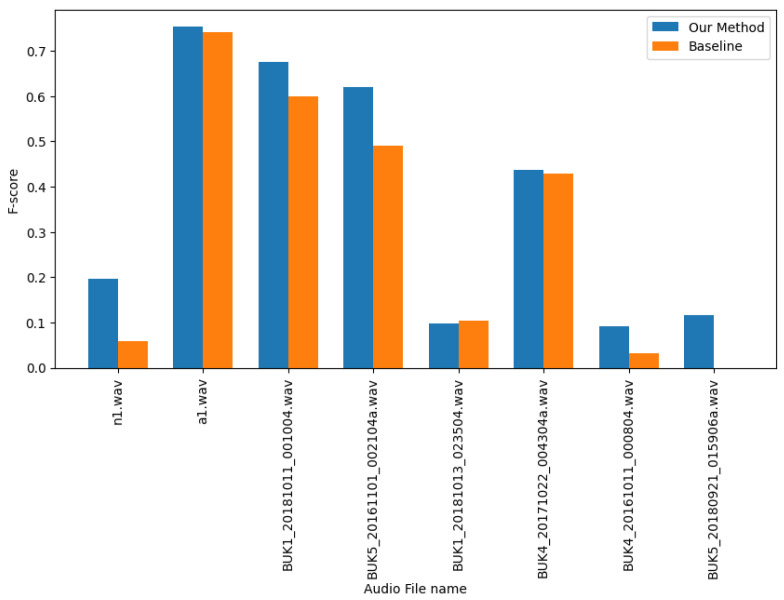
F-scores for audio recordings from the DCASE-2021 dataset.

**Figure 7:**
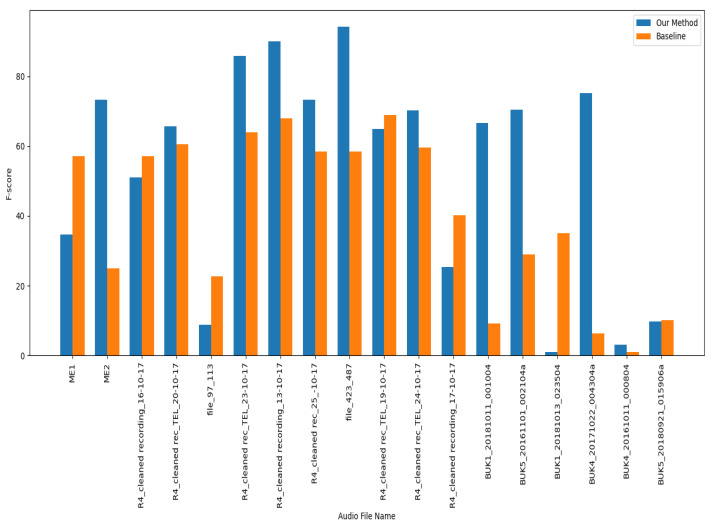
F-scores for audio recordings from the DCASE-2022 dataset.

The graphs in Figure 6 and Figure 7 show that some clean audio recordings have high-performance scores. However, the embedding space can become more scattered for more complex classes with domain noise, making them harder to predict accurately.

To better validate our results, we explored different FSL settings and compared the performance of our method against a baseline method. Table 9 and Table 10 present the performance under different FSL settings. The results indicate that our proposed method outperformed the baseline method at all FSL settings, demonstrating its effectiveness in solving the domain noise problem.

The proposed method for FS bioacoustic event detection employing transductive inference with data augmentation demonstrated promising results in enhancing performance scores. However, this approach is computationally complex and requires more resources than traditional methods. Transductive inference also requires a significant amount of unlabeled data to perform optimally. Additionally, generating diverse and representative augmented data using this method may not be well-suited to some audio recordings. Therefore, for practical applications in bioacoustic event detection, further research is necessary to address these limitations and refine this approach. It is possible to expand our research work in several ways to improve event detection performance. In terms of future work, integrating contextual features and ambient data with bioacoustic signals can greatly improve performance. Additionally, methodologies that require fewer unlabeled data, such as active learning and semi-supervised learning, are worth considering as potential alternatives to transductive inference.

## 4. Conclusions

This study investigated FSL techniques and applied them to bioacoustic event detection. Our research overcomes the challenge of limited labeled data within bioacoustic event detection by leveraging the knowledge gained from labeled and unlabeled data and data augmentation techniques. We modified the input spectrograms by employing frequency masking, temporal masking, and time warping. The goal of data augmentation is to boost model generalization by increasing the amount of labeled data. The input features used in the experiments were Mel spectrograms with PCEN. The experiments were conducted on DCASE datasets: DCASE-2021 and DCASE-2022. Various experimental settings were explored, and standard metrics for detecting bioacoustic events (F-score, precision, and recall) were used to evaluate the results. Overall, the proposed method outperformed the baseline system by a large margin. Our method effectively adapts to various animal species, recording environments, and durations, making it highly versatile for bioacoustic monitoring. Finally, the study highlights the importance of using bioacoustic event detection techniques for real-world scenarios, especially to protect biodiversity.

## Figures and Tables

**Table 1:** Parameters setting for Mel spectrogram feature extraction.

Sample rate	22.05 kHz
Segment length (seg_len)	0.04 s
Hop size	0.02 s
Number of points for Fast Fourier Transform (n_fft)	1024
Number of Mel filters (n_Mel)	128
Hop size for Mel spectrogram (hop_Mel)	256
Epsilon value for numerical stability (eps)	2.2204 $\times 10^{-16}$

**Table 2:** Information on the DCASE-2021 development datasets.

	Datasets	Mic Type	Audio Files	Total Duration	Labels (excl.UNK)	Events (excl.UNK)
	BirdVox	Fixed	5	10 h	11	2662
Training	Hyenas (HT)	Mobile	3	3 h	3	435
	Meerkats	Mobile	2	70 min	4	1234
	Jackdaws	Mobile	1	10 min	1	355
Validation	Hyenas (HV)	Mobile	2	2 h	2	50
	Polish Baltic	Fixed	6	3 h	2	260

**Table 3:** Information of DCASE-2022 development dataset.

	Datasets	Mic Type	Audio Files	Total Duration	Labels (excl.UNK)	Events (excl.UNK)
	BirdVox	Fixed	5	10 h	11	29,026
Training	Hyenas (HT)	various	5	5 h	5	611
	Meerkats	Mobile	2	70 min	4	1294
	Jackdaws	Mobile	1	10 min	1	357
	Wetlands Bird	various	161	5 h	26	2941
Validation	HumBug	Handheld	10	2.3 h	1	712
	Polish Baltic	Fixed	6	3 h	2	292
	Meerkats(ME)	Handheld	2	20 min	2	73

**Table 4:** Baseline results for the two DCASE datasets.

Datasets	Precision	Recall	F-Score
DCASE-2021	58.27%	32.2%	41.48%
DCASE-2022	36.34%	24.96%	29.59%

**Table 5:** Performance from various methods with DCASE-2022 and DCASE-2021.

Datatset	Method	F-Score	Precision	Recall
DCASE-2021	Prototypical Network	41.48%	58.27%	32.20%
	Anderson [46]	26.24%	20.00%	38.10%
	Hao Cheng [45]	45.96%	46.64%	46.28%
	TIM (ours)	49.85%	56.73%	44.46%
	**TIM + SpecAug (ours)**	51.64%	58.25%	46.38%
DCASE-2022	Prototypical Network	29.59%	36.34%	24.96%
	Li et al. [47]	47.88%	52.11%	44.30%
	Xiaoxiao and Long [48]	53.87%	64.20%	45.41%
	TIM (ours)	54.76%	55.34%	54.20%
	**TIM + SpecAug (ours)**	56.03%	59.93%	52.60%

**Table 6:** DCASE subset scores from employing our proposed method.

DCASE-2021			
**Subset**	**Precision**	**Recall**	**F-score**
Hyenas (HV)	50.83%	51.60%	49.62%
Polish Baltic	67.45%	41.03%	53.42%
**DCASE-2022**			
**Subset**	**Precision**	**Recall**	**F-score**
Meerkats (ME)	26.73%	92.30%	41.54%
HumBug	67.18%	77.64%	72.04%
Polish Baltic	44.94%	34.78%	39.21%

**Table 7:** DCASE-2022 scores per audio recording via the baseline and our method.

	Baseline			Ours		
**Audio File**	**Precision**	**Recall**	**F-Score**	**Precision**	**Recall**	**F-Score**
ME1	47.0%	72.7%	57.1%	21.9%	81.8%	34.6%
ME2	85.7%	14.6%	25.0%	57.7%	100%	73.2%
R4_cleaned recording_16-10-17	54.5%	60.0%	57.1%	37.5%	80.0%	51.0%
R4_cleaned rec_TEL_20-10-17	55.1%	67.1%	60.5%	67.2%	64.0%	65.6%
File_97_113	23.0%	22.5%	22.7%	19.4%	5.80%	8.9%
R4_cleaned rec_TEL_23-10-17	62.5%	65.4%	63.9%	93.0%	79.7%	85.8%
R4_cleaned recording_13-10-17	54.8%	89.4%	68.0%	85.7%	94.7%	90.0%
R4_cleaned rec_25_-10-17	54.8%	62.6%	58.4%	73.0%	73.7%	73.3%
File_423_487	54.8%	62.6%	58.4%	89.0%	100%	94.2%
R4_cleaned rec_TEL_19-10-17	63.2%	75.4%	68.8%	61.6%	68.5%	64.9%
R4_cleaned rec_TEL_24-10-17	56.6%	62.9%	59.6%	90.4%	57.5%	70.3%
R4_cleaned recording_17-10-17	62.1%	46.4%	53.1%	10.7%	8.30%	9.30%
BUK1_20181011_001004	8.00%	11.1%	9.30	78.2%	58.0%	66.6%
BUK5_20161101_002104a	26.3%	32.2%	28.9%	92.5%	56.8%	70.4%
BUK1_20181013_023504	48.9%	27.2%	35.0%	1 $\times \;10^{-3}$%	1 $\times \;10^{-3}$%	1 $\times \;10^{-3}$%
BUK4_20171022_004304a	14.2%	4.10%	6.40%	48.0%	70.5%	75.1%
BUK4_20161011_000804	1 $\times \;10^{-3}$%	1 $\times \;10^{-3}$%	1 $\times \;10^{-3}$%	5.80%	2.10%	3.10%
BUK5_20180921_015906a	12.5%	8.60%	10.2%	11.1%	8.60%	9.70%

**Table 8:** Scores per audio recording from the DCASE-2021 dataset.

Audio File	Baseline			Ours		
	**Precision**	**Recall**	**F-Score**	**Precision**	**Recall**	**F-Score**
n1.wav	4.00%	11.1%	5.80%	18.3%	22.2%	19.6%
a1.wav	70.0%	82.3%	74.1%	64.5%	90.9%	75.4%
BUK1_20181011_001004	78.9%	48.3%	60.0%	75.0%	61.3%	67.5%
BUK5_20161101_002104a	96.6%	32.9%	49.1%	97.5%	45.4%	62.0%
BUK1_20181013_023504	14.2%	8.30%	10.5%	11.8%	8.30%	9.80%
BUK4_20171022_004304a	54.5%	35.2%	42.8%	46.6%	41.1%	43.7%
BUK4_20161011_000804	7.10%	2.10%	3.20%	16.6%	6.30%	9.20%
BUK5_20180921_015906a	1 $\times \;10^{-3}$%	1 $\times \;10^{-3}$%	1 $\times \;10^{-3}$%	1.80%	8.60%	11.7%

**Table 9:** Performance under different settings with the DCASE-2022 dataset.

Method	Baseline (%)			Our (%)		
	**Precision **	**Recall**	**F-Score**	**Precision**	**Recall**	**F-Score**
5-way 5-shot	42.26	27.37	33.34	59.93	52.60	**56.03**
5-way 1-shot	16.43	24.49	19.66	39.81	52.42	**45.25**
10-way 5-shot	40.05	23.17	29.35	43.15	59.52	**50.22**
10-way 1-shot	12.88	24.78	17.05	37.95	46.68	**41.87**

**Table 10:** Performance under different settings with the DCASE-2021 dataset.

Method	Baseline (%)			Our (%)		
	**Precision **	**Recall**	**F-Score**	**Precision**	**Recall**	**F-Score**
5-way 5-shot	37.58	45.29	41.08	58.25	46.38	**51.64**
5-way 1-shot	40.15	24.21	30.21	39.50	36.56	**37.98**
10-way 5-shot	30.11	46.32	36.50	59.23	32.47	**41.95**
10-way 1-shot	15.93	38.01	22.45	28.18	40.25	**33.15**

## Data Availability

The original contributions presented in the study are included in the article, further inquiries can be directed to the first author.

## Abbreviations

The following abbreviations are used in this manuscript:

FSL	Few-Shot Learning
STL	Sound Transmission Loss
CNN	Convolutional Neural Network
PCEN	Per-Channel Energy Normalization
DCASE	Detection and Classification of Acoustic Scenes and Events
TI	Transductive Inference
ProtoNet	Prototypical Network
ResNet	Residual Network
